# Supplementary material for: Poor Sleep Quality Experience and Self-Management Strategies in Fibromyalgia: A Qualitative Metasynthesis
Source: J Clin Med. 2020 Dec 10;9(12):4000. doi: 10.3390/jcm9124000 (PMC7763602; doi:10.3390/jcm9124000)
Supplement: Supplementary file 1 [file jcm-09-04000-s001.pdf]

Table S1. poor sleep quality management strategies in FM.

OVERARCHING THEMES	SUBTHEMES-CODES	QUOTATIONS
		**Evaluation of poor sleep quality**
**EXPERIENCE OF POOR SLEEP QUALITY**	Poor sleep quality is a severe symptom of FM	*"It is very frustrating. I think the problem with sleeping is sometimes the worst of this."*<br>*"I can sleep I can go to off sleep but if I don't take anything, I do lie awake, just lie awake or I get woken up this constant being woken up is the worse thing"*<br>*"Sleep, or lack of it, is the worst thing about this condition for me […] It's just another way my body has betrayed me."*<br>*"Sleep is such a gift—I never take it for granted."*<br>*"I fell asleep fine, for an hour. Then I couldn't get back to sleep for I don't know how long."*<br>*"I don't feel like I can sleep. This is aging me; I can feel it. Sometimes I just hate life."*<br>*"I go to bed about 10, I'm always tired and I invariably 90% of the time go straight to sleep and I will wake 1–2 hours later feeling as if it is time to get up"*<br>*"Maybe five, yeah, four or five hours, and there's many times I wake up at 3:00, 4:00. And that's not enough…"*<br>*"I just know I wake up, and I-and I'm like, oh, toss and turn. And then I only sleep on my shoulder for maybe like 30 minutes."*<br>*"I can, initially, go to sleep, but it's staying asleep that's very hard. And then, I got to sleep tired and I wake up exhausted, and it's frustrating."*<br>*"I've become really restless throughout the night. I have trouble falling asleep. And then, I'm kind of in that dozed, as you described, state. You're never fully asleep. You're never fully awake. And I can toss and turn and just shift and move."*<br>*"You toss and turn, you can't keep still, I'm not being funny but my legs have spasms, they just jump around like someone's putting an electric shock through you every 5 seconds, your limbs jump and twitch"*<br>*"A typical night if you can describe the sleep as typical, is I go to bed around 10, 10:30, I fall asleep straight away (clicks fingers) drop of a hat, head on the pillow and I'm away, I'll sleep for 2 or 3 hours, then I'm up for between 2 or 3 hours, then I'll sleep for 2 hours and then I'm up"*<br>*"I am so tired most of the time, that I feel that I could nap at any time. However, I don't sleep for long and I am awake again."*<br>*"I said I used to sleep really well. I could sleep all the way through, like before I was diagnosed with fibromyalgia or, you know, even having all the symptoms. I could sleep through the storm. Now anything will wake me up"*<br>*"And then you're staying in bed. I pretty much stay in bed all day"*
	Perceived effects of poor sleep quality in other symptoms of FM	*"I think that the pain is one thing that wears you out probably the biggest thing. I know it is a good part of the reason I don't sleep well"*<br>*"I sometimes get so tired that I can't go to sleep. I ache everywhere, and my muscles do a little dance number"*<br>*"I have worked about 47 hours this week, and I have been in so much pain from stress and overwork that I couldn't sleep all week"*<br>*"I don't hardly ever sleep with my husband anymore, because I disturb his sleep so much of the time with my tossing and turning, trying to get comfortable, getting in and out of bed, because I can't get comfortable"*<br>*"If I sleep really well at night, I'm in much worse shape in the morning as far as pain goes. If I'm up and down all night long, I'm not in as much pain because I've been moving. But then I'm a lot more tired, so I'm not going to be doing all that much"*<br>*"Pain leads to a bad night, and a bad night leads to pain the next day"*<br>*"I've quite often woke up feeling more tired than when I went to bed"*<br>*"There is no typical day, I wake up and it depends on what kind of sleep I've had, how broken and how bad a night I've had"*<br>*"In some ways it's more difficult because I'm so tired I don't have the energy to cope with things, so I think they get me down a bit more"*<br>*"'Lack of sleep affects your pain and er, and if you're in a lot of pain and you roll over, the pain will wake you up"*<br>*"And you can't get comfortable, you know…. you can never get comfortable; your neck is never in the right position…"*

	"Even if I do get a good night's sleep, let's assume like six hours, a good night's sleep, and I wake up at 6 in the morning, I can't function to do anything until 12:00 noon. I can't do the dishes, I can't do anything, it's like you're just-you're lethargic, you know"<br>*"If I don't get enough sleep that will cause a flare-up, but it also causes me to not be able to sleep at the same time."*<br>*"I could sleep 20 hours and still be tired. That is terrible."*<br>*"But when you sleep, it doesn't resolve it. You still wake up tired"*<br>*"The fatigue, it's number one, because I can deal with the pain, at least up to a certain point, but the fatigue there's nothing you can do besides sleep. There is no way to help that. There's no pill you can take, there's no medicine."*<br>*I will be thinking and, and trying to explain stuff to you, but my mind will just go completely blank. That gets worse on certain days, obviously with less sleep, but on other days I can sort of string together"*<br>*"Having a bit more energy, say, after a good night's sleep, I've got a bit more energy to be able to go a whole day and to do things; after a bad night's sleep fatigue will hit me at say half 3 in the afternoon eventually, plug's pulled and I fall asleep standing up more or less"*<br>*"Sometimes when I've had a bad night my appetite goes as well. I have to eat something to take my medication, but I will force myself to eat a bit of toast or something, you know, just so I've got something in my tummy to take the tablets"*<br>*"After a bad night's sleep, my muscles and my joints can be really quite painful and tight cause I haven't rested them properly"*<br>*"It's not just the pain and the fatigue, . . . it's the nonrestorative sleep . . . it's a vicious circle because if you don't get enough sleep you feel pain more acutely . . . you're more tired and unable to sleep well"*<br>*"I conserve energy whenever possible. Problems can arise, though, no matter how well you plan; the pain, fatigue, or irritable bowel syndrome episodes prevent you from doing anything. Sometimes all three attack at once, when you might have been fairly okay an hour ago. Even if you try to get a good sleep, that doesn't mean you can have any energy on waking, and just because you baby yourself . . . doesn't mean you won't hurt. Coping is minute-to-minute every single day"*<br>*"Sleeplessness nights ... can you see the black circles under my eyes, because I wake up at 2 o'clock in the morning, then I wake up and feel right awake...and the next day I feel tired, I just want to sleep on but I have to get up and go to work ... I am extremely physically tired ... I am so tired I just can't function as a normal person"*<br>*"Yes, you never feel as you have slept enough. Sometimes I sleep very well¼ but you are never thoroughly rested when you wake up in the morning"*
Beliefs about the temporality and cause of poor sleep quality	*"I don't know what other reason there could be, other than the fact that I'm less active now than I used to be you know, but you know it seems to have come on roughly about the same time"*<br>*"[After a night shift] I slept maybe an hour or hour and a half a day … you know how it is when you don't sleep practically at all for weeks and months … In fact, I was practically sleepless for years …"*
Meaning of good sleep quality	*"It's that sensation of really I have switched off, I am not aware of anything. That you know, those three hours where maybe the following day my husband said to me, "Oh did you hear the thunderstorm last night?" "No," because it happened on those three hours and I didn't hear anything. I didn't hear the thunderstorm, I didn't notice the light, nothing, and that is for me a proper sleep. When I'm aware of everything else I'm not, and I get up noticing that I have not slept properly"*<br>*"I know when I've had a good night's sleep because I would wake in the morning feeling refreshed"*
	**Response to poor sleep quality**
Feeling frustrated and like a failure	*"I hate this illness! I hate not being able to stand the sun, the heat, feeling tired all the time, not being able to sleep. The list goes on of things I hate about this illness"*<br>*"It is very frustrating. I think the problem with sleeping is sometimes the worst of this"*<br>*"The not sleeping and then not being able to function the next day when you need to perform at work . . . when you're being paid and you're meant to work and you can't function, it's horrible, it's really horrible because you feel like a failure"*

		"All I know is, is that I never used to need a lot of sleep, never, and I now really resent that fact that I need so much, I'm not saying my life is so exciting but when it's, just now I think oh, I have to plan it round, oh can I have a lay in this weekend, can I do this, can I do that, I find that very hard to cope with"<br>*"I can, initially, go to sleep, but it's staying asleep that's very hard. And then, I got to sleep tired and I wake up exhausted, and it's frustrating"*<br>*"I don't feel like I can sleep. This is aging me; I can feel it. Sometimes I just hate life."*<br>*Night was described by some as a "lonely" time, and again the impact of not being able to sleep on day to day functioning was described as "an absolute nightmare"*
	Fear of going to bed	*"Sleep, or lack of it, is the worst thing about this condition for me. I have christened my bedroom "the torture chamber"*<br>*"If there's something on my mind, that makes me a bit worried about sleeping, I don't know what it is but I sort of need to try and get to the bottom of it, but I sort of have this fear of going to bed"*<br>*"We're wanting to start a family, so that's kind of an issue regarding sleep, actually; yes, that's a massive issue, because we've been wanting to start a family for ages, we're not even doing that because I don't think I could have that sort of sleep deprivation"*<br>*"If there's something on my mind, that makes me a bit worried about sleeping, I don't know what it is but I sort of need to try and get to the bottom of it, but I sort of have this fear of going to bed"*<br>*"If you're worried about one of the children or there's something particular you're worried about..., you tend to sort of go over that in your mind"*<br>*"You almost know, that's psychological again, you almost know you've had a fairly bad period you're not going to sleep well either or is it a fact?"*
**POOR SLEEP QUALITY MANAGEMENT STRATEGIES IN FM**		**Management strategies to favor sleep**
	Medication: from dependency to rejection	*"I ache almost continually, that is why I take three aspirin every night before I go to bed. When I try to skip it, I end up getting up in an hour or two and taking them anyway."*<br>*"Well, I cannot sleep at all if I don't have [medication], so. . .there is no sleeping without it. . . And getting up in the morning was just brutal. It's brutal. I have to drag my sorry butt out of bed every morning and go to work, and it's hard"*<br>*"I can sleep I can go to off sleep but if I don't take anything, I do lie awake, just lie awake or I get woken up this constant being woken up is the worse thing"*<br>*"I suppose it helps a little bit but I wouldn't say it makes a tremendous difference you know, I suppose I'd rather take it than take nothing, any help is better than no help, but I still find myself waking up quite frequently every night"*<br>*Using sleeping pills was usually not seen as an option because of side-effects, such as "foggy mornings"*<br>*"I am on Amytriptiline to help with my sleep and other tablets... I only take them when it is right for my body. I don't like to be taken as a guinea pig. I don't trust staff to deal with me in that way. I am the only person who knows what it feels like to be ill, and what is good for my body, I don't like other people to tell me and to control me"*
	Self-management: behavioral adaptations	*"I tend not to watch TV too late unless there's something really good on but I try and make an effort, like I used to really like watching DVDs late at night and now I don't do that as much or I'll listen to the radio, it's a bit more chilled really, yeah drink herb tea just sort of wind down"*<br>*"All I know is, is that I never used to need a lot of sleep, never, and I now really resent that fact that I need so much, I'm not saying my life is so exciting but when it's, just now I think oh, I have to plan it round, oh can I have a lay in this weekend, can I do this, can I do that, I find that very hard to cope with"*<br>*"I know that it's an absolute crucial thing to get a good night's sleep; to go to bed at the same time; eating on time... so everything has to be regular. And the more regular it is, the better I feel"*<br>*"I'll try anything to help me relax, deep breathing and visualization, anything to make me go to sleep. But nothing works"*<br>*"I put ear plugs in and I find when I put ear plugs in I seem to sleep different . . . although I don't like things in my ears, if it means I'm going to sleep a bit better, so whether that's about noise, although it's very quiet where I live, so I don't know, I think I'm just looking for answers"*<br>*"Sometimes I think I can get back to sleep, so I wait to see if I can and I think come on and I just lie there and hopefully I can get back to sleep easily again, um, but more often than not, I can't so after I try for about 15 to 20 minutes and if I can't get back to sleep after that time then as I say I put the television on and it'll refocus me and if I fall back to sleep, good"*

	"Oh I just get up, it's just ridiculous trying to sleep and you can't sleep, you know you get to hate your bed after a while because you get fed up tossing around and trying to get to sleep the whole time"
**Managing the consequences of a sleepless night**	
Medication: finding the balance between benefits and side effects	*"I try to get out of bed every day regardless of how I feel. I'm trying to function, so I take the various medications . . . in the hopes that while it causes other problems it will at least allow me to continue to have a life"*<br>*"[…] When I have been resting a while and I sleep when I've taken a painkiller… and can sleep an hour and then it feels much better, then I can manage the afternoon"*
Resting and relaxing during the day	*"I've been overworked, overstressed, and overtired. When I am fatigued I sleep, and that's what I've been doing in my spare time"*<br>*''I'm at a place now where I, when I get tired, I say okay. And I'll go and lie down. And I may nap for half an hour, one hour, or three hoursy"*<br>*"If I can get sleep, I can fix all the rest"*<br>*"It can be up to an hour, at least an hour, even if I didn't sleep, I just have to get off my legs and just lie back and you know, very often I just lie back with nothing on, no television, nothing, just, just alone"*<br>*"I might have to go to bed for a couple of hours and then I'll be alright for the evening, because I know they advise you not to go to bed don't they, but I can't physically not and I find it makes me feel better actually if I do, so for me it works better, so you I've learnt to do what suits me rather than what I'm told to do you know they say you muck up your body clock up if you sleep in the day but for me it doesn't work that way"*<br>*"…lavender smell. Yes. So you're getting the olfactory there and it's got the, Epson salt-type stuff in it and you know, whatever. And it just, a warm bath sometimes will get me so I get over that stiffness, when I can't get out of bed…"*<br>*"You just have to lie down and rest, you can't do anything, can't go on or anything… it passes when you rest a moment… that total exhaustion […]"*
